# Supplementary material for: Non-invasive regional cerebral blood flow quantification in the 123I-IMP autoradiography using artificial neural network
Source: PLoS One. 2023 Mar 8;18(3):e0281958. doi: 10.1371/journal.pone.0281958 (PMC9994717; doi:10.1371/journal.pone.0281958)
Supplement: Attachment — Submitted filename: Response to Reviewers.docx [file pone.0281958.s002.docx]

We thank the reviewer very much for the kind and expert review. We revised our manuscript along the comments.

**For Reviewer #1’s comments:**

**1. Please kindly explicitly mention the hyperparameters used for the ANN mode in-text. I did notice it mentioned in the figure, kindly move the information to the methods section.**

- Thank you for your comment. We described the hyperparameters used for the ANN in the first paragraph of methods section. We kindly ask for your confirmation.

**2. There is a discrepancy in the following statement:**

**"In this study, the Bayesian optimization method was performed to automatically optimize the hyperparameters of an ANN model, including the number of hidden layers, number of neurons in the hidden layer, and activation functions in a network [14]. However a few sentence later the author claimed the following: "The number of hidden layers and Epoch were manually set to 3 and 30, respectively. "Kindly confirm if it was selected or was it optimized?**

- We sincerely apologize for the inconvenience caused by our mistake. As you mentioned, the discrepancy occurred about decision procedure for the number of hidden layers. In the present study, the number of hidden layers was manually determined, not automatically optimized by Bayesian optimization method. Therefore, we deleted the phrase "the number of hidden layers" from the sentence about the Bayesian optimization method. We kindly ask for your confirmation.

-----

Additionally, we revised our manuscript along with PLOS ONE's style requirements and described the full name of IRB.
