# Supplementary material for: Non-invasive regional cerebral blood flow quantification in the 123I-IMP autoradiography using artificial neural network
Source: PLoS One. 2023 Mar 8;18(3):e0281958. doi: 10.1371/journal.pone.0281958 (PMC9994717; doi:10.1371/journal.pone.0281958)
Supplement: Attachment — Submitted filename: Response to Reviewers.docx [file pone.0281958.s003.docx]

We thank the reviewer and editor very much for the kind and expert review. We revised our manuscript along the comments.

**For Editor‘s comments:**

**Thank you for addressing the question - although your reply was inaccurate, however the changes in the manuscript was reflected accurately.　Please also check the following.**

**Page 9 - Fig6s or Fig6a?**

- Thank you for your comment. The description **“Fig 6s”** in Page 9 line 16 was correct. We described **“Fig 6s”** as including Fig 6a to 6f, but it could be confusing. We changed the description “**Fig 6s”** to simply “**Fig 6”.** For the same reason, the description **“Fig 5s”** in Page 9 line 2 was changed to “**Fig 5”**. If you have any other opinion or intention, please let me know.

In addition, we added supporting information S1 including raw data sets used for machine learning.
